# Neuroprotective effects of kojic acid and nano-kojic acid in experimental brain ischemia induced by bilateral common carotid artery occlusion

**DOI:** 10.22038/ijbms.2025.89790.19367

**Published:** 2026

**Authors:** Mohammad Shokati Sayyad, Majid Saeedi, Fatemeh Shaki, Fereshteh Talebpour Amiri, Reza Negarandeh, Mohammad Seyedabadi

**Affiliations:** 1 Department of Toxicology and Pharmacology, Faculty of Pharmacy, Mazandaran University of Medical Sciences, Sari, Iran; 2 Pharmaceutical Sciences Research Center, Institute of Herbal Medicines and Metabolic Disorders, Mazandaran University of Medical Sciences,; 3 Sari, Iran; 4 Department of Pharmaceutics, Faculty of Pharmacy, Mazandaran University of Medical Sciences, Sari, Iran; 5 Department of Anatomy, Faculty of Medicine, Mazandaran University of Medical Sciences, Sari, Iran; 6 Cellular and Molecular Biology Research Center, Health Research Institute, Babol University of Medical Sciences, Babol, Iran

**Keywords:** Anti-oxidants, Inflammation, Ischemia, Neuroprotection, Oxidative stress

## Abstract

**Objective(s):**

Brain ischemia remains a leading cause of death and neurological disability worldwide, with current treatments limited by narrow therapeutic windows and insufficient neuroprotection. Kojic acid (KA), a natural compound with demonstrated anti-oxidant and anti-inflammatory properties, has not been thoroughly evaluated in cerebral ischemia. Its limited brain bioavailability may have hindered the identification of its potential therapeutic effects. This study aimed to investigate the effects of both KA and its nanostructured lipid carriers (nKA) in a rat bilateral common carotid artery occlusion (BCCAO) model.

**Materials and Methods:**

Adult male rats were randomly allocated to seven groups: sham, BCCAO, KA (1 and 10 mg/kg), nKA (1 and 10 mg/kg), and vehicle. Following BCCAO surgery, animals received intraperitoneal treatments for seven days. Behavioral assessments included the modified neurological severity score and grid walk test. Brain and serum samples were collected to evaluate histopathology, gene expression, oxidative stress markers, inflammatory cytokines, and pharmacokinetic parameters.

**Results:**

Histological analysis indicated reduced neuronal loss in both KA and nKA-treated groups. Notably, only nKA significantly improved behavioral outcomes. Both treatments reduced the pro-apoptotic gene BAX and the pro-inflammatory cytokine TNF-α, while nKA additionally increased Nrf2 and reduced IL-6 expression. Both formulations mitigated oxidative stress by decreasing reactive oxygen species, protein carbonylation, and malondialdehyde levels, while also increasing glutathione concentrations.

**Conclusion:**

Nano-kojic acid showed better neurobehavioral improvements, while both forms reduced oxidative stress and inflammation, indicating neuroprotective potential. Further studies are needed to clarify mechanisms and long-term safety.

## Introduction

Stroke, defined as a sudden interruption or significant reduction of blood and oxygen supply to a localized region of the brain, remains a leading cause of mortality and long-term disability worldwide (1). It is broadly categorized into ischemic and hemorrhagic subtypes, with ischemic stroke accounting for the majority of cases and associated with higher mortality rates (2). One of the primary contributors to ischemia is occlusion of the carotid arteries, whether unilateral or bilateral, which results in neuronal loss and disruption of normal brain functions (3). Clinically, these events present as deficits in motor coordination and cognition, and in severe cases, can be fatal. Without adequate preventive measures and therapeutic interventions, projections estimate that ischemic stroke-related mortality could reach approximately 7.8 million deaths globally by 2030 (4, 5). 

The pathophysiology of ischemic stroke is highly complex, involving a cascade of biochemical and cellular events. The initial deprivation of glucose and oxygen leads to energy failure, impaired ATP synthesis, and ionic imbalance, which in turn induces excitotoxicity, mitochondrial dysfunction, and ultimately, cell death (6, 7). A hallmark of this process is the overproduction of reactive oxygen species (ROS) and reactive nitrogen species (RNS), such as superoxide, hydrogen peroxide, and nitric oxide, which drive oxidative stress and disrupt the delicate oxidant-anti-oxidant balance in neural tissue. This imbalance results in lipid peroxidation, protein oxidation, DNA damage, and further mitochondrial impairment (8, 9), as reflected by elevated biomarkers including malondialdehyde (MDA) and protein carbonyls (PC), and depletion of endogenous anti-oxidants like glutathione (GSH). The Nrf2/HO-1 signaling pathway is a critical endogenous defense mechanism, regulating the expression of cytoprotective genes and playing a key neuroprotective role during ischemia-reperfusion injury (9-11). 

Concomitantly, neuroinflammation is initiated by damage, releasing pro-inflammatory cytokines such as tumor necrosis factor-alpha (TNF-α) and interleukin-6 (IL-6). This inflammatory cascade promotes blood-brain barrier (BBB) disruption and further neuronal damage (12, 13). Additional markers such as lactate dehydrogenase (LDH) and nitric oxide further indicate ongoing tissue injury and inflammation. The interplay between pro-apoptotic (BAX) and anti-apoptotic (BCL2) proteins also critically determines neuronal survival following ischemic insult (14-16).

 Under physiological conditions, the brain relies on a network of anti-oxidant defense systems, including both enzymatic and non-enzymatic mechanisms, that counteract the damaging effects of ROS and maintain redox homeostasis. However, when these protective systems are overwhelmed or impaired, oxidative damage accumulates, leading to neuronal dysfunction and increased vulnerability to neurodegeneration (17, 18).

The bilateral common carotid artery occlusion (BCCAO) model is widely used in rodents to mimic human cerebral ischemia or ischemic stroke, providing a reproducible and clinically relevant platform for investigating the mechanisms of ischemic injury and evaluating potential therapeutic interventions. While current pharmacological strategies for ischemic stroke focus primarily on restoring blood flow, there is a growing recognition of the need for adjunctive therapies that target the secondary injury cascades of oxidative stress and inflammation, which are not adequately addressed by reperfusion alone (19, 20). So, two primary pharmacological strategies are employed in ischemic stroke: reopening blocked blood vessels and protecting against ischemia-reperfusion injury. The latter is particularly important as it involves inflammatory cascades and oxidative stress triggered by various gene and molecular factors, upon reoxygenation of the ischemic cells, leading to tissue damage (21). 

Kojic acid (KA), a natural secondary metabolite first isolated from Aspergillus oryzae, has gained considerable attention for its potent anti-oxidant and anti-inflammatory properties. As a metal chelator, KA inhibits ROS generation and lipid peroxidation, thereby mitigating oxidative stress (22, 23). Preclinical studies have demonstrated that KA can reduce oxidative stress markers, enhance the expression of Nrf2 and HO-1, and suppress neuroinflammatory pathways, including the inhibition of TNF-α and IL-1β production. These effects have been observed in models of neuroinflammation and neurodegeneration, where KA administration led to reductions in ROS and lipid peroxidation, an increase in cytoprotective genes, and improved functional outcomes. These mechanistic insights provide a compelling rationale for evaluating KA as a neuroprotective agent in ischemic stroke, where both oxidative and inflammatory cascades are central to neuronal injury (24, 25). These findings provide a strong mechanistic rationale for evaluating KA in the context of cerebral ischemia, where both oxidative and inflammatory cascades are prominent contributors to neuronal injury.

Despite these promising preclinical findings, the therapeutic application of KA in cerebral ischemia has been limited by its poor bioavailability and restricted brain penetration. Nanoparticle-based drug delivery systems, such as nano-kojic acid (nKA) formulations, have been developed to overcome these challenges by enhancing pharmacokinetic properties, tissue distribution, and targeted delivery (26-28). Encapsulation within nanoparticles can improve the stability and solubility of KA, potentially amplifying its beneficial effects on oxidative stress and inflammation in the context of cerebral ischemia. 

By these considerations, the present study aims to investigate the neuroprotective effects of KA and its nano-formulation in a rat model of cerebral ischemia induced by BCCAO. We employ a comprehensive approach, including biochemical and histological analyses, evaluation of key gene pathways, assessment of behavioral changes related to inflammation and oxidative stress, and quantification of drug levels in the brain using high-performance liquid chromatography (HPLC). 

## Materials and Methods

### Chemicals

KA was obtained from Sigma Aldrich (United Kingdom, USA). Acetonitrile and ketamine were sourced from Merck (Germany), and xylazine was purchased from Alfasan (Woerden, Netherlands). All other chemicals used in this study were of analytical grade and utilized without further purification. 

### Animals

Fifty-six male Wistar rats, weighing 200–220 g and aged 12–13 weeks, were obtained from the Animal Laboratory of Mazandaran University of Medical Sciences (Sari, Iran). Animals were housed under standard laboratory conditions with ad libitum access to food and water. All experimental procedures were approved by the Committee of Animal Experimentation at Mazandaran University of Medical Sciences and conducted in accordance with the National Institutes of Health Guide for the Care and Use of Laboratory Animals (IR.MAZUMS.AEC.1403.018). 

### Experimental design and animal groups

The rats were randomly assigned to seven groups, with eight animals in each group, as outlined in Table 1. Brain ischemia was induced using a standardized protocol, and animals received daily intraperitoneal injections of KA, nKA acid, or vehicle. On the seventh day following brain ischemia induction, behavioral assessments, including the Grid Walk test and the Modified Neurological Severity Score (mNSS), were performed to evaluate motor coordination and neurological function. On the eighth day, rats were deeply anesthetized and sacrificed in the Institute for Experimental Animal Research of our university. After confirming the absence of reflexes for deep pain, the animals were beheaded, the skull was immediately opened, and brain tissues were collected for analyses of oxidative stress markers, gene expression, and HPLC quantification. At the same time, serum samples were prepared for subsequent biochemical evaluations. The samples were stored as mentioned in each section.

### Induction of brain ischemia (cerebral ischemia/reperfusion)

Brain ischemia was induced in rats using the BCCAO technique, as previously validated in preclinical studies. Animals were anesthetized by intraperitoneal injection of ketamine (75 mg/kg) and xylazine (10 mg/kg). A midline cervical incision (~1 cm) was made to expose both common carotid arteries, identified by their distinct pulsatile morphology. The arteries were isolated from adjacent vagal tissue and bilaterally occluded with microvascular clamps for 20 min to induce transient global ischemia. Subsequent clamp removal restored cerebral reperfusion, after which the surgical site was closed with sutures. Postoperative treatments were administered intraperitoneally for seven consecutive days, with tissue and serum samples collected immediately after behavioral tests. Sham-operated controls underwent identical surgical exposure without arterial occlusion, ensuring procedural equivalence while maintaining baseline cerebral perfusion (3, 29).

### Synthesis of nano-kojic acid

The nanostructured lipid carriers (NLCs) containing KA are fabricated based on a melt emulsification followed by the ultrasonication technique. Briefly, the lipid phase consisting of cholesterol, oleic acid, glyceryl monostearate (GMS), and Span^®^ 60 was heated to 90–95 °C until fully melted, then mixed evenly. In a separate beaker, the aqueous phase, which contains deionized water and Tween^®^ 20, was mixed and then heated to the same temperature as the oily phase. Subsequently, two-thirds of the aqueous phase with KA is added dropwise to the lipid phase under high-speed stirring to form a pre-emulsion. The mixture then undergoes 5 min of 100% amplitude probe sonication (Bandelin, 3100, Germany). After the first sonication period, the remaining one-third of the water phase in an ice-water bath was added to the mixture, which was then quickly placed in the bath. Finally, the mixture was sonicated for 5 min and allowed to cool at room temperature (30).

### Small-animal PET imaging (Micro PET)

Cerebral glucose metabolism was assessed using a small-animal PET scanner (Xtrim PET) at the Preclinical Core Facility (TPCF) of Tehran University of Medical Sciences, Iran. Before imaging, rats underwent an 8-hour fasting period with ad libitum water access. Under general anesthesia (ketamine/xylazine), animals received a tail vein injection of 18F-fluorodeoxyglucose (18F-FDG; 0.7 mCi) and were allowed a 60-minute awake uptake period to minimize anesthesia-related metabolic interference. Imaging was performed in the prone position using a dedicated rodent bed, with raw data reconstructed into coronal, sagittal, and axial planes (31, 32).

### Behavioral tests

To evaluate behavioral and motor impairments seven days after BCCAO-induced brain ischemia, two established tests were employed: The Grid Walk Test and the Modified Neurological Severity Score.

Grid Walk Test: The Grid Walk Test, also known as the foot-fault test, is a widely used method to assess motor coordination and sensorimotor deficits in rodent models of stroke. In this test, rats were allowed to freely traverse a 45 cm × 45 cm grid. As the animals moved, the number of foot placement errors (foot faults) was recorded. The score was calculated as the percentage of foot errors relative to the total number of footfalls, providing an objective measure of limb coordination and motor impairment. Healthy rats typically exhibit few foot faults, whereas ischemic rats display a marked increase in errors, reflecting deficits in motor control and coordination (33, 34).

Modified Neurological Severity Score: The mNSS is a comprehensive scoring system designed to evaluate neurological function in rodent stroke models, incorporating assessments of motor ability, sensory response, balance, and reflexes. The scale ranges from 0 to 18, where a score of 0 indicates normal neurological function and 18 represents severe impairment. Higher scores correspond to greater neurological deficits, enabling sensitive detection of functional changes following ischemic injury (35). 

### Histopathological examination

Brain tissues were fixed in 10% formalin for 24 hr to preserve morphological integrity and facilitate histopathological evaluation. Following fixation, samples underwent standard tissue processing, which included sequential dehydration in graded ethanol solutions, clearing with xylene, and embedding in paraffin wax. Sections of 5 μm thickness were then prepared and stained using hematoxylin and eosin (H&E) for general histological assessment, as well as cresyl violet (Nissl staining) to evaluate neuronal integrity. Stained slides were examined under a light microscope (Olympus, Japan) to assess histopathological changes (36). 

### Oxidative stress assay

Oxidative stress parameters were assessed in brain tissue samples using established biochemical methods. Initially, protein concentration was determined by the Bradford assay with Coomassie Blue reagent (37). Levels of ROS were quantified using dichlorodihydrofluorescein diacetate (DCFH-DA) as a fluorescent probe, with measurements performed on a fluorometer (Jasco, Japan; model FP6200) at excitation and emission wavelengths of 480 nm and 520 nm, respectively. GSH content was measured in tissue homogenates using DTNB reagent, and absorbance was read at 405 nm with a microplate reader (37). Lipid peroxidation was evaluated by determining MDA levels, employing thiobarbituric acid as an indicator and measuring absorbance at 545 nm (38). PC content was assessed by treating samples with 2,4-dinitrophenylhydrazine (DNPH) and recording absorbance at 370 nm (39). Myeloperoxidase (MPO) activity was determined using a commercial kit (Nampox, Navand Salamat Co., Iran), based on the reaction of tetramethylbenzidine with hydrogen peroxide. The absorbance was measured at 650 nm (38). All assays were conducted according to the manufacturer’s protocols or established literature methods, and results were normalized to protein concentration.

### Inflammatory markers (NO and LDH)

Tissue nitric oxide levels were measured using the Natrix kit (Navand Salamat Co., Iran), which quantifies NO indirectly by detecting nitrite formed from the reaction with sulfanilic acid, resulting in an azo dye measured at 570 nm using a microplate reader (39). This colorimetric approach is widely used in research for assessing NO and its metabolites in various biological samples, providing a reliable estimate of tissue NO content. Serum levels of lactate dehydrogenase were determined biochemically using the Pars Azmoon kit and analyzed with a Hitachi 902 autoanalyzer. 

### Gene expression

Gene expression analysis was performed to quantify the mRNA levels of TNF-α, IL-6, BAX, BCL2, NRF2, and HO-1 in rat brain tissue. Following euthanasia, brain samples were immediately placed in RNA shield solution (DENA Zist Asia, Iran) and stored at -80 °C to preserve RNA integrity. Total RNA was extracted using the Pars Toos Total RNA Extraction Kit (Pars Toos Company, Mashhad, Iran) based on the silica column method, ensuring high yield and purity as recommended for reliable downstream applications. The concentration and quality of RNA were assessed spectrophotometrically (Agilent Technologies, USA) at 230, 260, and 280 nm, and further confirmed by agarose gel electrophoresis with documentation (Biorad, USA). Complementary DNA (cDNA) was synthesized from the extracted RNA using the Pars Toos cDNA Synthesis Kit (Pars Toos Company, Iran) in a PCR thermocycler (Biorad, USA). Quantitative real-time PCR (qRT-PCR) was then conducted on a Corbett Rotorgene 6000 instrument (Qiagen, Germany) using gene-specific primers and a defined thermal cycling protocol. Glyceraldehyde 3-phosphate dehydrogenase (GAPDH) was used as the endogenous reference gene to normalize expression levels of the target genes, following established best practices for accurate normalization in qRT-PCR studies (39). Relative gene expression was calculated using the comparative 2^ -^^ΔΔCT^ method as detailed in the Table 2.

### Quantification of drug levels in tissue samples

Brain tissue samples were homogenized in 2 ml of normal saline using a homogenizer operating at 10,000 RPM to ensure thorough extraction of the drug. The homogenate was centrifuged at 10,000 RPM for 20 min to separate cellular debris, and the resulting supernatant was mixed with an equal volume of absolute ethanol to precipitate proteins. After vortexing for two minutes, the mixture was centrifuged again under the same conditions, and the clear supernatant was filtered through a syringe filter to remove any remaining particulates. The prepared samples were then analyzed by HPLC using a Knauer Smartline system (Germany) equipped with UV detection set at 268 nm. Separation was achieved on a reverse-phase C18 column (250 mm × 4.6 mm) using a modified mobile phase composed of 80:20 (v/v) 0.1% acetic acid and acetonitrile, delivered at a flow rate of 1 mL/min over a total run time of 10 min. This method enabled precise quantification of the drug concentration in brain tissue by comparing sample peak areas to those of calibration standards, thereby determining the percentage of drug present in the homogenized brain samples. 

### Statistical analysis

All data were analyzed using one-way analysis of variance (ANOVA), followed by Tukey’s *post hoc* test for multiple comparisons. Statistical analyses were performed with Prism software (version 9). Results are expressed as mean ± standard error of the mean (SEM), and a *P*-value of less than 0.05 was considered statistically significant.

## Results

### Small-animal PET imaging

**Figure 1:**
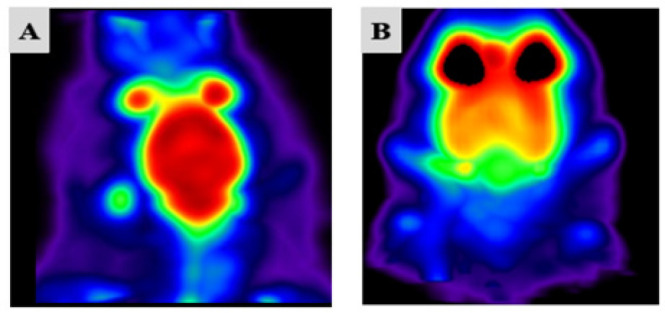
Representative micro-PET images depicting alterations in cerebral glucose metabolism in rat brains

To evaluate brain status and validate our experimental procedures, we employed micro-positron emission tomography imaging to assess cerebral activity in both the sham (non-ischemic) and model (cerebral ischemia) groups. Micro-PET scans were performed one hour after intravenous administration of 18F-fluorodeoxyglucose (18F-FDG) by the tail vein. This technique enabled visualization of regional glucose metabolism in the brain. In the sham group, areas of high metabolic activity were indicated by red and yellow regions, reflecting robust glucose uptake. In contrast, the BCCAO model group exhibited green and blue regions, corresponding to reduced glucose utilization and hypometabolism following stroke induction. These findings demonstrate diminished glucose uptake and metabolic activity as characteristic consequences of cerebral ischemia and injury (Figure 1). 

### Behavioral tests

**Figure 2:**
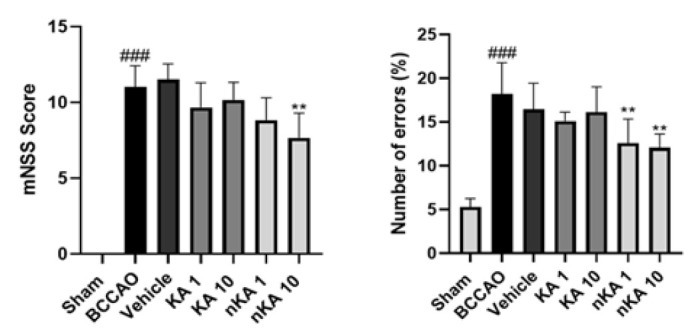
(A) Assessment of neurological function using the modified neurological severity score (mNSS) in rats

To evaluate changes in behavioral and neurological function, the Grid Walk and mNSS tests were conducted following the final drug treatment. After cerebral ischemia induction in the model group, both assessments revealed a significant increase in impairment and a reduction in normal motor and behavioral function compared to the sham group (### *P*<0.001 vs sham). The vehicle-treated group exhibited results comparable to the model group, with no significant differences observed. Administration of nKA at 10 mg/kg led to a significant improvement, as evidenced by a reduction in mNSS scores (** *P*<0.01 vs BCCAO group). NKA1 and nKA10 notably decrease error percentage in the Grid Walk test (** *P*<0.01 vs BCCAO group). Although the groups treated with KA at 1 and 10 mg/kg showed a trend toward improvement in both tests, these changes did not reach statistical significance compared to the model group (Figure 2).

### Histopathological tests (Hematoxylin-Eosin and Nissl Staining)

Histopathological evaluation using hematoxylin-eosin (H&E) and cresyl violet (Nissl) staining revealed distinct patterns of brain tissue injury and recovery across experimental groups following BCCAO and subsequent treatments. In coronal sections of the cortex, the sham group displayed well-preserved tissue architecture with clearly defined cell boundaries and nuclei, and neurons appeared morphologically intact, consistent with the absence of ischemic injury. In contrast, the BCCAO group exhibited pronounced histopathological alterations, including the presence of pigmented and shrunken neurons, indicative of acute ischemic damage.

**Figure 3:**
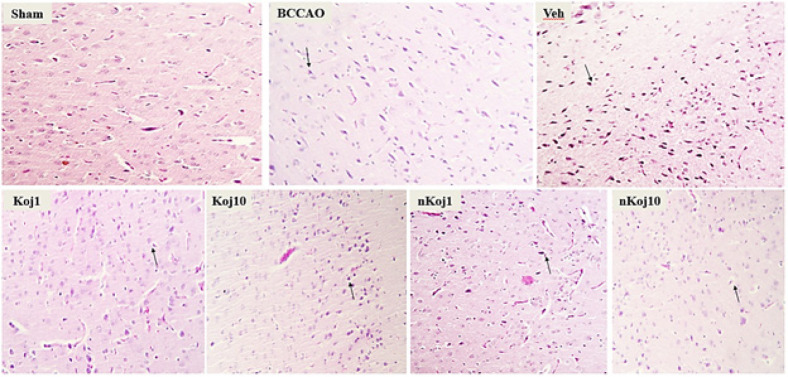
Representative hematoxylin and eosin (H&E) stained sections illustrating morphological alterations in rat brain tissue following BCCAO surgery

**Figure 4:**
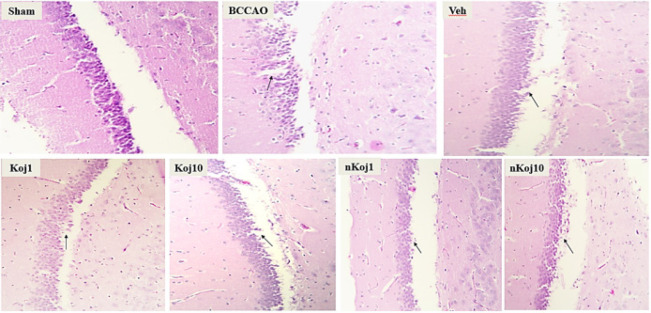
Representative hematoxylin and eosin (H&E) stained images showing morphological alterations in rat brain tissue across experimental groups

**Figure 5:**
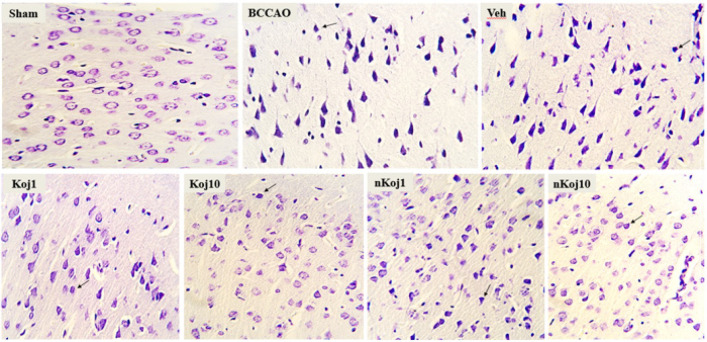
Representative cresyl violet (Nissl) stained sections illustrating morphological changes in rat brain tissue across experimental groups

Treatment with KA at both 1 and 10 mg/kg resulted in partial preservation of tissue structure compared to the untreated model group, as highlighted by regions with normal-appearing cells and areas containing degenerated or pigmented neurons (black arrow) in Figure 3. Notably, groups receiving nKA (1 and 10 mg/kg) demonstrated further improvement in tissue integrity, particularly in the hippocampus, where reduced neuronal degeneration and improved histological appearance were observed relative to the BCCAO group (Figure 4). Nissl staining further corroborated these findings: the sham group showed abundant neurons with intact morphology, while the BCCAO and vehicle groups exhibited marked neuronal loss, decreased nuclear density, and disrupted tissue architecture characterized by pyknotic nuclei, shrunken cytoplasm, and increased extracellular space. In contrast, the nKA 10 mg/kg group showed better preservation of neuronal structure and overall tissue integrity compared to the model group, as depicted in Figure 5.

### Oxidative stress markers

Findings related to oxidative stress markers in brain tissue were assessed. In all tests, the BCCAO group was first compared with the sham group, indicated by the # symbol. This was followed by comparisons of the other five groups with the BCCAO group, denoted by the star symbol (*). Additionally, the KA and nKA groups were compared with the sham group, with a lack of significant difference indicated by the caret (^) symbol. 

Assessment of oxidative stress markers in brain tissue following BCCAO revealed significant alterations consistent with ischemic injury. ROS levels were markedly increased in the model group compared to the sham group. Treatment with KA (10 mg/kg) and nKA (10 mg/kg) significantly reduced ROS levels relative to the model group. GSH, a key non-enzymatic anti-oxidant, was depleted considerably in both the model and vehicle groups compared to the sham group. Administration of KA (1 mg/kg) and nKA (1 and 10 mg/kg) significantly increased GSH levels compared to the model group. MDA, a marker of lipid peroxidation and oxidative injury, was significantly elevated in the model group. Both the nKA (1 and 10 mg/kg) and KA (10 mg/kg) groups demonstrated significant reductions in MDA levels compared to the model group, indicating reduced lipid peroxidation.

PC content, an indicator of protein oxidation, was also significantly higher in the model group versus the sham group. Treatment with KA (1 and 10 mg/kg) and nKA (1 mg/kg) resulted in significant decreases in PC levels compared to the model group. However, the KA (1 mg/kg) and nKA (1 mg/kg) groups showed no significant difference from the sham group.

**Figure 6:**
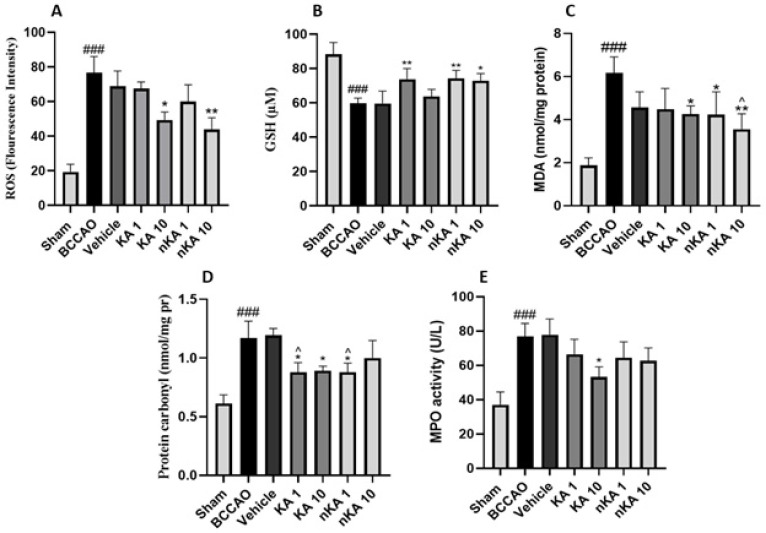
Alterations in oxidative stress and antioxidant biomarkers among rat experimental groups

Finally, MPO activity, which reflects neutrophil infiltration and oxidative inflammatory response, was significantly increased in the model groups compared to the sham group, consistent with established models of BCCAO-induced neuroinflammation and oxidative stress. Only the KA (10 mg/kg) group showed a significant reduction in MPO activity compared to the model group, while other treatment groups did not exhibit significant changes. Overall, these results demonstrate that BCCAO induces marked oxidative stress in the brain, as evidenced by increased ROS, MDA, PC, and MPO activity, and decreased GSH levels. Treatment with KA and nKA, particularly at higher doses, effectively mitigated these oxidative disturbances (Figure 6).

### Inflammatory markers (NO and LDH)

**Figure 7:**
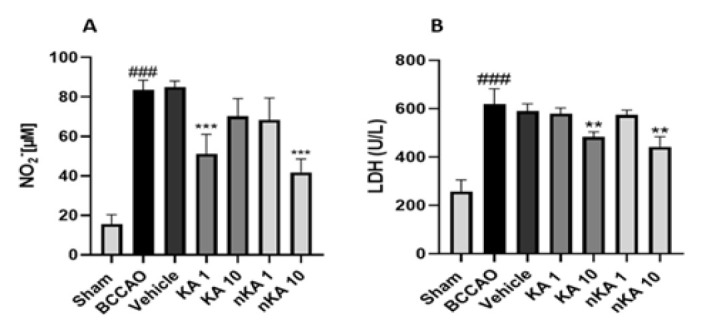
Serum levels of (A) nitric oxide (NO) and (B) lactate dehydrogenase (LDH) across all rat experimental groups

Assessment of inflammatory markers revealed significant alterations following BCCAO-induced cerebral ischemia. Indirect measurement of NO levels in brain tissue demonstrated a marked elevation in both the model BCCAO groups compared to the sham group. Treatment with KA at 1 mg/kg and nKA at 10 mg/kg resulted in a significant reduction in NO levels relative to the model group (A). Similarly, LDH concentrations were significantly higher in the model group than in the sham group. At the same time, administration of KA 10 mg/kg and nKA 10 mg/kg significantly decreased LDH levels compared to the model group (B). These findings indicate that both KA and its nano-formulation, particularly at higher doses, can attenuate key inflammatory markers following cerebral ischemia (Figure 7). 

### Gene expression

**Figure 8:**
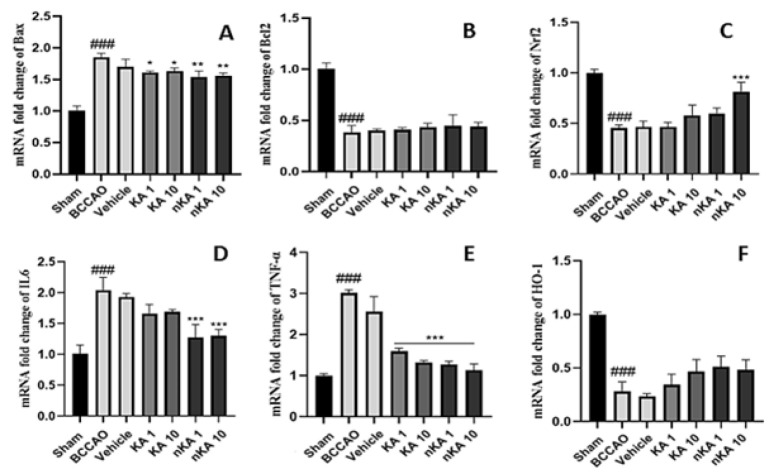
Relative mRNA expression levels of key apoptotic, anti-oxidant, and inflammatory markers in rat brain tissue

Gene expression profiling in rat brain tissue demonstrated significant alterations among the experimental groups (Figure 8). Compared to the sham group, the BCCAO model group showed a marked increase in pro-apoptotic (BAX) and pro-inflammatory (IL-6, TNF-α) genes, with a significant decrease in anti-apoptotic (BCL2) and anti-oxidant (NRF2, HO-1) genes (### *P*<0.001). Administration of nKA acid at both 1 and 10 mg/kg doses resulted in significant reductions in IL-6 and TNF-α expression, while treatment with KA at 1 and 10 mg/kg also significantly decreased TNF-α levels (***P<0.001). Notably, an increase in NRF2 expression was observed only with the higher dose (10 mg/kg) of nKA (***P<0.001). In contrast, no significant differences were detected among the treatment groups for BCL2 and HO-1 gene expression. KA and nKA acid treatment did not significantly alter BCL2 or NRF2 expression. Additionally, the vehicle group did not exhibit significant changes compared to the BCCAO model group. These findings highlight the modulatory effects of KA and its nano-formulation on key apoptotic, inflammatory, and anti-oxidant gene pathways following cerebral ischemia. 

### Quantification of drug levels in tissue samples

**Figure 9:**
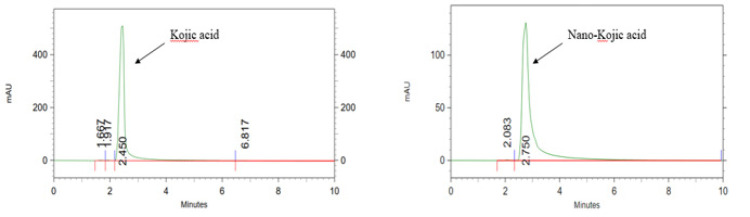
Representative high-performance rat liquid chromatography (HPLC) chromatograms of kojic acid (KA) and nano-kojic acid (nKA), monitored at 268 nm

Quantitative analysis of KA in rat brain tissue was performed using HPLC, demonstrating the method’s capability for specific and sensitive detection of both KA and its nano-formulation. Distinct chromatographic peaks for KA and nKA were observed at retention times of 2.45 and 2.75 minutes, respectively (Figure 9). No significant interfering peaks were detected at these retention times, confirming the assay’s specificity for the analytes under the established chromatographic conditions. Following administration, the percentage of KA detected in brain tissue was 12.59% at a dose of 1 mg/kg and 4.63% at 10 mg/kg, while nKA was present at 17.9% for 1 mg/kg and 6.27% for 10 mg/kg. Importantly, this reduction in percentage does not imply a lower absolute concentration of the drug in the brain. In fact, our data show that with increasing doses, the actual concentration of the drug in the brain tissue increased, though in a non-linear fashion.

## Discussion

Brain ischemia remains a leading cause of mortality and long-term disability worldwide, primarily resulting from cerebral hypoperfusion or vascular occlusion. Despite advances in ischemic stroke care, therapeutic options remain limited and are often associated with adverse effects, underscoring the urgent need for innovative neuroprotective strategies to mitigate ischemic injury and promote functional recovery (40-42). The bilateral common carotid artery occlusion model serves as a well-characterized preclinical system for studying global cerebral ischemia, where both the ischemic event and subsequent reperfusion trigger distinct yet interconnected injury cascades. Central to this pathophysiology lies a pathological triad of oxidative stress, neuroinflammation, and apoptotic signaling mechanisms that drive neurodegeneration, manifest behaviorally as cognitive-motor deficits and potentially fatal outcomes (43-45). These pathological processes collectively contribute to neuronal loss, cognitive-motor deficits, and increased mortality, emphasizing the need to target multiple injury pathways to achieve neuroprotection.

KA (5-hydroxy-2-hydroxymethyl-4H-pyran-4-one), a fungal-derived organic compound, is best known for its skin-lightening effects through tyrosinase inhibition. It also exhibits additional pharmacological benefits, including anti-oxidant, antibacterial, anti-inflammatory, and leukocyte-modulating activities. Despite these recognized properties, the detailed mechanisms behind its multifaceted biological actions are not yet fully elucidated (28, 46). It exhibits low acute toxicity in rodents, with an oral LD₅₀ in rats exceeding 1000 mg/kg body weight, supporting its safety at gram-per-kilogram doses (47). Preclinical studies in rodent models have demonstrated its neuroprotective effects against neuroinflammation and oxidative stress. Doses around 50 mg/kg elicit anti-inflammatory responses without reported toxicity in short-term administration (48). To further inform our dose selection, we conducted pilot studies to gain insights into the effective dose range and potential toxicity, confirming minimal adverse effects at lower doses. 

In the present study, BCCAO induced significant cerebral hypometabolism, elevated oxidative stress markers, and increased neuroinflammatory cytokine levels in rats, accompanied by behavioral impairments and histopathological evidence of brain injury. Treatment with both KA and its nanoformulation conferred neuroprotective effects. Notably, nKA at 10 mg/kg produced significant improvements in motor coordination and neurological function, whereas conventional KA showed trends toward behavioral recovery that did not reach statistical significance. Histopathological analysis revealed enhanced neuronal preservation with both treatments, especially at higher doses. Regarding oxidative stress biomarkers, both KA and nKA at 10 mg/kg reduced ROS, while increases in GSH were observed in the KA1, nKA1, and nKA10 groups, consistent with enhanced endogenous anti-oxidant capacity. MDA and protein carbonyl content were reduced in KA10, nKA1, and nKA10. Inflammatory biomarkers such as TNF-α and IL-6 were significantly decreased, particularly in nKA-treated animals. Treatment with nKA10 resulted in significant reductions in LDH and NO levels, all recognized markers of tissue damage and systemic inflammation. The KA10 group also exhibited reductions in LDH. Importantly, MPO activity was not affected by nKA treatment, probably indicating that the beneficial effects of nKA are independent of MPO-related mechanisms. 

Molecular analysis revealed an increase in Nrf2 expression in the nKA10 group and a reduction in the pro-apoptotic protein BAX in all treatment groups, though anti-apoptotic BCL2 and HO-1 levels remained unchanged. HPLC confirmed brain penetration of both KA and nKA, corroborating their central bioavailability. Collectively, these data suggest that KA, as well as its nanoformulation, mitigated cerebral ischemic injury primarily through anti-oxidant and anti-inflammatory mechanisms.

These findings support and extend previous research demonstrating the neuroprotective effects of KA in experimental models of oxidative stress and neuroinflammation. Oral administration of KA has been shown to reduce LPS-induced neuroinflammation by improving cognitive function and suppressing inflammatory mediators such as TNF-α and IL-1β. Moreover, KA treatment enhances Nrf2 gene expression. It decreases ROS levels, consistent with observed reductions in oxidative stress markers (ROS, MDA, PC) and inflammatory cytokines (TNF-α) in KA- and nKA-treated groups. However, in our study, the conventional form of KA did not induce a significant change in Nrf2 gene expression, which may be attributed to several factors, including variations in species (mice vs rats) and dosage regimens (49).

Furthermore, KA was shown to attenuate inflammation and restore impaired autophagy by suppressing NF-κB and PI3K/AKT/mTOR signaling in an osteoarthritis model. This finding highlights a mechanism through which KA modulates cellular homeostasis under oxidative stress, a pathway potentially relevant to ischemic brain injury given the critical role of autophagy in neuronal survival (50). Our observed reductions in apoptotic marker BAX and oxidative damage support a similar anti-apoptotic effect in cerebral ischemia. 

Khan *et al*. (2021) further corroborated KA’s neuroprotective role in an amyloid-β (Aβ)-induced Alzheimer’s disease mouse model. They demonstrated decreased oxidative stress, lipid peroxidation, and neuroinflammation through the inhibition of TLR4/NF-κB signaling and pro-inflammatory cytokines, alongside improved synaptic function and memory. These results parallel our findings, including brain bioavailability confirmed by HPLC, reinforcing the translational potential of KA and its nanoformulation for neurodegenerative conditions characterized by oxidative and inflammatory pathology (24). Beyond neurological diseases, KA exhibits broad-spectrum anti-inflammatory and antimicrobial activities. Montazeri *et al*. (2019) reported that KA reduced parasitic load and tissue damage in experimental toxoplasmosis by modulating host immune responses, suggesting that its immunomodulatory effects may be broadly applicable in diverse inflammatory contexts (23). Additionally, KA administration mitigated hematological and oxidative damage in radiation-exposed animals, further emphasizing its anti-oxidative versatility (25, 51). Studies in reproductive biology provide mechanistic insights into KA’s anti-apoptotic properties. Hu *et al*. (2024) showed that KA inhibited apoptosis in porcine spermatozoa by enhancing mitochondrial anti-oxidant defenses, decreasing cleaved caspase-3 and caspase-9 (52). These findings correspond with our observations of reduced BAX expression and oxidative stress in KA/nKA-treated ischemic brains. Kotyzová *et al*. (2004) also reported that KA mitigated oxidative damage and regulated trace element homeostasis in iron-overloaded rodent models, a relevant mechanism given the role of iron-mediated oxidative injury in cerebral ischemia (53). Together, these studies reinforce the multi-modal anti-oxidant and anti-apoptotic actions of KA that likely underlie its neuroprotective efficacy. 

Advances in nanotechnology-based brain drug delivery support the superior efficacy of nKA in our study. A recent comprehensive review highlights NLCs as second-generation lipid nanocarriers, offering improved biocompatibility and drug-loading capacity. They overcome the limitations of traditional delivery systems and provide targeted CNS delivery (54-56). This supports our findings that nKA surpasses conventional KA in behavioral assays, although their effects are relatively comparable in molecular tests. Nonetheless, we observed only a slight increase in brain penetration upon nano-formulation. 

The biological activities of KA, including its anti-oxidant and anti-inflammatory effects, are attributable to its chemical structure. The hydroxyl groups on the pyrone ring facilitate hydrogen donation to neutralize free radicals, while metal chelation prevents Fenton reaction-mediated ROS generation (28). These mechanisms align with our findings, where KA and nKA reduced oxidative damage and inflammation in cerebral ischemia.

While our findings in general indicate a neuroprotective role for KA against cerebral ischemia, especially when formulated in NLC, a clear dose-response relationship was not observed in this study. This may be explained by receptor saturation, off-target effects, divergent endogenous inputs to the dependent variables of study, and non-linear kinetics (57-60). In particular, we did not find a 10-fold increase in brain concentration of KA when we increased the dose by 10 times in either of the conventional or NLC formulations. Our data show that with increasing doses, the actual concentration of the drug in the brain tissue increased, though in a non-linear fashion. For example, at 1 mg/kg of KA, the brain concentration was 0.311 µg/mg protein (12.59%). At 10 mg/kg, the concentration increased to 1.14 µg/mg protein, although the percentage decreased to 4.63%. Similarly, for nKA, the concentration increased from 0.443 µg/mg protein (17.9%) at 1 mg/kg to 1.550 µg/mg protein (6.27%) at 10 mg/kg. These findings highlight a non-linear dose-concentration relationship, which is common in CNS drug delivery and nanoparticle-based formulations.

Our study indicates that KA and its nKA significantly reduce oxidative stress markers, including ROS, MDA, and PC, as well as pro-apoptotic BAX and pro-inflammatory cytokines IL-6 and TNF-α, in a BCCAO model of cerebral ischemia in rats. These findings suggest that the neuroprotective effects of KA and nKA are likely mediated through attenuation of oxidative stress and neuroinflammation, with nKA potentially benefiting from enhanced brain penetration due to its formulation. However, the precise molecular pathways and their relative contributions remain incompletely understood, necessitating further studies to dissect the specific roles of these mechanisms through selective pathway inhibition. Comprehensive investigations are required to optimize dosing regimens and evaluate the long-term safety and efficacy of KA and nano-KA for potential clinical translation in stroke management. 

**Table 1:** Animal groups (8 rats in each group)

Group	Description
Sham	Control (without ischemia and drug)
Model (BCCAO)	Brain ischemia (without drug)
KA1 mg/kg	Brain ischemia + KA 1 mg/kg
KA 10 mg/kg	Brain ischemia + KA 10 mg/kg
nKA 1 mg/kg	Brain ischemia + nKA 1 mg/kg
nKA10 mg/kg	Brain ischemia + nKA 10 mg/kg
Vehicle	Brain ischemia + vehicle

**Table 2:** The sequence of primers and thermal conditions used for the analysis of gene expression in rats

Genes	Primer	Thermal condition
TNF-α	F: TCAGCCTCTTCTCATTCCTGCT R: GCCATTGAACTGATGAGAGGGA	95 °C, 5 min 40 cycles (95 °C, 20 sec; 63 °C, 20 s; 72 °C, 20 sec) 72 °, 5 min
IL-6	F: TATACCACTTCACAAGTCGGAGG R: GAATTGCCATTGCACAACTCTTT	95 °C, 5 min 40 cycles (95 °C, 20 sec; 63 °C, 20 s; 72 °C, 20 sec) 72 °, 5 min
Bcl-2	F: CTGTGGATGACTGAGTACCTGA R: AGCCAGGAGAAATCAAACAGAGG	95 °C, 5 min 40 cycles (95 °C, 20 sec; 63 °C, 20 sec; 72 °C, 20 sec) 72°, 5 min
Bax	F: CCAAGAAGCTGAGCGAGTGT R: TGTCCAGCCCATGATGGTTC	95 °C, 5 min 40 cycles (95 °C, 20 sec ; 65 °C, 20 sec; 72 °C, 20 sec) 72°, 5 min
NRF2	F: ACATCCAGACAGACACCAGTGG R: GGAATGTCTCTGCCAAAAGCTG	95 °C, 5 min 40 cycles (95 °C, 20 sec; 66 °C, 20 sec; 72 °C, 20 sec) 72°, 5 min
HO	F: CACAGCACTACGTAAAGCGTCT R: GTAGCGGGTATATGCGTGGG	95 °C, 5 min 40 cycles (95 °C, 20 sec; 63 °C, 20 sec; 72 °C, 20 sec) 72°, 5 min
GAPDH	F: 5´CCCCCAATGTATCCGTTGTG 3´ R: 5´TAGCCCAGGATGCCCTTTAGT3´	95 °C, 5 min 40 cycles (95 °C, 20 sec; 62 °C, 20 sec; 72 °C, 20 sec) 72°, 5min

(A) Sham group: rats that did not undergo bilateral common carotid artery occlusion surgery. (B) BCCAO group: rats subjected to the cerebral ischemia model via BCCAO, with imaging performed seven days post-procedure. BCCAO: Bilateral common carotid artery occlusion; PET: Positron Emission Tomography

###*P*<0.001 compared to the sham group; **P<0.01 compared to the BCCAO group. (B) Evaluation of motor coordination by the grid-walking test. ###*P*<0.001 compared the sham group; **P<0.01 compared the BCCAO group

Statistical analysis was performed using one-way ANOVA followed by Tukey’s* post hoc *test. Data are presented as mean ± standard deviation (SD). BCCAO: Bilateral common carotid artery occlusion

The Sham group received normal saline without cerebral ischemia induction; the BCCAO group underwent bilateral common carotid artery occlusion; the Vehicle group received vehicle plus BCCAO; the KA1 and KA10 groups were treated with 1 mg/kg and 10 mg/kg kojic acid plus BCCAO, respectively; the nKA1 and nKA10 groups received 1 mg/kg and 10 mg/kg nano-kojic acid plus BCCAO, respectively. Black arrows indicate pyknotic cells characterized by condensed nuclei and cell shrinkage. Images were captured at 200× magnification, with a scale bar representing 100 μm. BCCAO: Bilateral common carotid artery occlusion

The Sham group received normal saline without cerebral ischemia induction; the BCCAO group underwent bilateral common carotid artery occlusion; the Vehicle group was treated with vehicle plus BCCAO; the KA1 and KA10 groups received 1 mg/kg and 10 mg/kg kojic acid plus BCCAO, respectively; the nKA1 and nKA10 groups were administered 1 mg/kg and 10 mg/kg nano-kojic acid plus BCCAO, respectively. Black arrows indicate a lower number of cells compared to the sham group and the presence of pyknotic cells. All images were captured at 200× magnification, with a scale bar indicating 100 μm.

The Sham group received normal saline without BCCAO induction; the BCCAO group underwent bilateral common carotid artery occlusion; the Vehicle group was administered vehicle plus BCCAO; the KA1 and KA10 groups received 1 mg/kg and 10 mg/kg kojic acid plus BCCAO, respectively; and the nKA1 and nKA10 groups were treated with 1 mg/kg and 10 mg/kg nano-kojic acid plus BCCAO, respectively. Black arrows indicate pyknotic cells characterized by condensed nuclei and cell shrinkage. All images were captured at 400× magnification, with a scale bar representing 100 μm.

(A) Reactive oxygen species (ROS), (B) glutathione (GSH), (C) malondialdehyde (MDA), (D) protein carbonyls (PC), and (E) myeloperoxidase (MPO) levels were quantified in all groups. The Sham group received normal saline without ischemia induction; the BCCAO group underwent bilateral common carotid artery occlusion without treatment; the Vehicle group was administered vehicle for 7 days post-BCCAO; the KA1 and KA10 groups were treated with 1 mg/kg and 10 mg/kg kojic acid intraperitoneally for 7 days after BCCAO, respectively; and the nKA1 and nKA10 groups received 1 mg/kg and 10 mg/kg nano-kojic acid intraperitoneally for seven days following BCCAO, respectively. Data are presented as mean ± standard deviation (SD) for each group.

###: *P*<0.001 significantly different from the sham group; *: *P*<0.05, significantly different from the BCCAO group; **: *P*<0.01 significantly different from the BCCAO group; ^: *P*<0.05, not significantly different from the sham group.

The Sham group received normal saline without brain ischemia induction; the BCCAO group underwent bilateral common carotid artery occlusion without treatment; the Vehicle group was administered vehicle following BCCAO; the KA1 and KA10 groups received 1 mg/kg and 10 mg/kg kojic acid intraperitoneally after BCCAO, respectively; and the nKA1 and nKA10 groups received 1 mg/kg and 10 mg/kg nano-kojic acid intraperitoneally for 7 days following BCCAO, respectively. Data are presented as mean ± standard deviation (SD) for each group.

Data are expressed as mean ± standard deviation (SD) for each group.###: *P*<0.001 significantly different from the sham group, **:* P*<0.01 compared with the BCCAO group, ***: *P*<0.001 compared with the BCCAO group

(A) bax, (B) bcl2, (C) nrf2, (D) IL-6, (E) tnf-α, and (F) HO mRNA fold changes are shown for each experimental group. The Sham group received no cerebral ischemia induction; the BCCAO group underwent bilateral common carotid artery occlusion without treatment; the Vehicle group received vehicle following BCCAO surgery; the KA1 and KA10 groups were treated with 1 mg/kg and 10 mg/kg kojic acid intraperitoneally after BCCAO, respectively; the nKA1 and nKA10 groups received 1 mg/kg and 10 mg/kg nano-kojic acid intraperitoneally after BCCAO, respectively. Data are presented as mean ± standard deviation (SD) for each group.###: *P*<0.001 significantly different from the sham group; *: *P*<0.05 compared with the BCCAO group; **: *P*<0.01 compared with the BCCAO group; ***: *P*<0.001 compared with the BCCAO group

Kojic acid eluted at a retention time of 2.45 min, while nKA exhibited a slightly delayed retention time of 2.75 min, reflecting formulation-dependent pharmacokinetic properties. The validated method demonstrated high analytical sensitivity, with a limit of detection (LOD) of 8.36 ng/mL and a limit of quantification (LOQ) of 27.89 ng/mL, ensuring precise quantification of both compounds in neural tissue matrices.

## Conclusion

This study demonstrates that nKA exhibits superior efficacy compared to conventional KA in improving neurobehavioral outcomes following the BCCAO model in rats. Meanwhile, the effects of KA in other tests are comparable to those of the nKA. The protective effects can be attributed to the intrinsic anti-oxidant and anti-inflammatory properties of KA, as well as a marginal increase in brain penetration upon nanoformulation. Our findings show significant attenuation of oxidative stress markers and suppression of pro-inflammatory cytokines, supporting the therapeutic potential of the chemical as an adjunctive neuroprotective agent in cerebral ischemia management. Nevertheless, given the complexity of its underlying mechanisms, further in-depth studies are warranted to elucidate its precise mechanisms of action and assess chronic safety.

## Data Availability

Data are available from the corresponding author upon reasonable request.

## Declaration

We acknowledge the use of Perplexity AI to enhance the language and readability of the manuscript. All data analyses and the preparation of figures and graphs were carried out independently, without the use of artificial intelligence.
